# Lectures on Clinical Medicine, Delivered at the Philadelphia Medical Institute

**Published:** 1838-07-18

**Authors:** W. W. Gerhard

**Affiliations:** Physician to the Philadelphia Hospital, &c.


### CLINICAL LECTURES.

LECTURES ON CLINICAL MEDICINE, *delivered at the Philadelphia Medical Institute, by* W. W. GERHARD, M. D., *Physician to the Philadelphia Hospital,* &c.

### GASTRITIS.

*Tuesday, June 12th.*—I shall now enter upon the subject of inflammations of particular mucous membranes. In my general lecture I have already stated that in these inflammations, you must not expect the same regular series of symptoms, that occur in the inflammations of other tissues; nor can you always detect with certainty their existence. The appearances they present after death are, likewise, often deceptive. Just as the redness, dependent on an erysipelatous condition of the skin, disappears after death, so may the traces of inflammation of the mucous membranes. You cannot, then, trust, entirely, even to pathological anatomy, to ascertain its presence, although the thickening, and other appearances which I detailed in a former lecture, are usually sufficient to enable you to arrive at a tolerably satisfactory conclusion.

To study properly the symptoms of inflammations of the mucous membranes, we cannot do better than follow the plan, hitherto employed, of carefully examining the history and symptoms of particular cases. For this purpose, I shall now bring before your notice a case, recently under treatment at the hospital, of severe gastritis, brought about by excessive intemperance. It is that of William Rogers, in ward No. 11., an Englishman by birth, and resident in this country for nineteen years. He has been living for five months, on the West-Chester road, and was well until within the last three weeks, when he was taken ill, with loss of appetite, and irregular chills, but without pain across the stomach. He was drinking, during the Easter holidays, and continued the frolic for four weeks. The vomiting began only a week before his entrance,—he had previously felt anorexia and nausea, but no pain. He was slightly costive; there had been no delirium, but some confusion of the intelligence. For three days before his admission, he had been vomiting blood in large quantities, which continued till the moment of his entrance. There was no previous treatment; nor was the mind of the patient sufficiently clear for him to recollect other symptoms than those just detailed. He is subject to fits, and had several on the day of his entrance, besides those which will be afterwards mentioned.

You see here, that the powers of the stomach, which had been over-stimulated by alcohol, gave way—but without much complication of cerebral symptoms. There was vomiting of pure bile, indicating gastritis, and afterwards of blood, which, as there was no cough or disease of the chest, we ascertained to be a genuine hematemesis, and not hemoptysis,—it was a simple effusion from the mucous membrane of the stomach. The symptoms, therefore, which were present, previous to the man's entrance, denoted severe inflammation of the stomach. At the time of his entrance on the 14th of May, they were as follows:—

The skin was pale and yellowish; not much emaciation. Eyes extremely prominent; pupils small; dulness of expression; mouth strongly compressed, but no distortion. No cephalalgia; giddiness, and, at times, a little ringing in both ears. Intelligence regular, but feeble. Skin dry and harsh. Pulse 104, feeble and small. There was no soreness in the epigastrium, nor any tumor in the region of the stomach. I examined particularly if there was a tumor, because hematemesis is often a sign of a cancerous affection of the stomach, which, in an advanced stage, can be detected by percussion and by the touch. There was constant eructation; bowels open, except for the last two or three days. Constant nausea, and discharge by vomiting of a liquid, of the colour of Spanish brown, slightly curdled, apparently altered blood without smell, and coming up with very little effort. No cough. Milk and water was ordered for drink, in small quantities.

| | | |
|---|---|---|
| ℞. | T. Opii, | gtt. v. |
| | T. Cinnamoni, | gtt. x. |
| | Sodæ Bicarbonat. | gr. x. |
| | Aquæ Cinnamoni, | ℥ss. f. sol. |
| M. | | |

Sig. To be taken every half hour.

No ice, or remedy of this kind, was ordered, because the patient had been accustomed to stimulate the mucous membrane of his stomach with a large daily allowance of spirits. Now in these cases the very best remedy is usually a stimulant, of a less irritating nature than that to which he has been accustomed.

On the fifteenth, the man vomited, seven or eight times, a thick curdy liquid, like gruel, without blood. At two, P. M., he began to take ice, at frequent intervals, and vomited occasionally after it, but less frequently. The eructations continued. No stools. Same feebleness, and pale, waxen colour of the skin. A blister was applied to the abdomen, at two, P. M. The mixture was discontinued, but from mistake a few more doses were taken.

On the sixteenth, the vomiting was much less frequent, having occurred only once or twice since the morning, and consisted chiefly of the liquid swallowed,—probably from a very slight admixture of blood, it was of the colour of Madeira wine and water. The man had taken no food. Same stupor; but the intelligence clearer, and he complained only of drowsiness; felt the blister. Less nausea; same paleness and sallowness of the countenance; anorexia and constipation continue. Convulsive fits without distortion of the mouth. Pulse 92, tolerably full. Skin of the hand warm. Ice continued. A tea-spoonful of brandy every half hour, and, if desired, a biscuit or two soaked in brandy and water. A common injection.

On the eighteenth, no tenderness in the abdomen, except externally. Dulness of the intellect less. Tongue moist and clean. No vomiting. Bowels twice open. Less stupor. Appetite returning. Pulse 80, soft and small. Skin of the natural temperature. The quantity of brandy given was reduced to two ounces a day. Ice continued.

On the twentieth, ice had been continued occasionally; two ounces of brandy were given each day. Four crackers taken, without vomiting; and a little gruel on the nineteenth. Still, belching of wind. Strength rather greater. Less stupor. Same complexion and fixed expression, with widely opened, staring eyes. Appetite returning; no abdominal soreness. Pulse 76, regular. Diet increased to six crackers and more gruel.

Twenty-first, same expression of countenance; pupils strongly contracted. No vomiting or purging. Abdomen rather hard, tympanitis. No tenderness at the epigastrium. Pulse 80, rather full. Brandy suspended. Infusion of ginger; crackers, gruel.

Twenty-third, no vomiting; great feebleness. One or two stools in twenty-four hours. No nausea; tympanitis. Appetite better; skin cool. Began yesterday a grain of quinine and ginger every three hours. Convalescence was more established.

Diarrhœa began on the twenty-eighth; had five or s ixstools, without griping, thin, yellow, and watery, without blood. No chill. No assignable cause, except the change in the weather, which became much warmer. Took quinine until the twenty-ninth, when the oleaginous mixture and a few doses of the chalk mixture were given him, and he is now free from diarrhœa.

Discharged, June 11th. Convalescence uninterrupted after last date. No return of diarrhœa; eructation continued; appetite good. Strength increasing. A vacant expression of countenance remained.

Now, we have here an example of a mild type of gastritis. The vomiting was the main symptom here, and, usually observed in most cases, is not necessarily present in every variety of gastritis. It may occur in very slight forms of the affection, and, on the other hand, erosion of the mucous membrane of the stomach may take place, without the existence of vomiting. In tolerably severe cases, it is generally present, and it will be found a very certain symptom of gastritis of moderate intensity; it may be wanting if the gastritis be exceedingly slight, or of great severity, nervous excitability, in the latter instance, being extinguished by the intense inflammation.

The absence of pain, in this case, can readily be accounted for, by the complication of epilepsy. Certain symptoms, belonging to the regular train of every disease, you may expect to find absent, in all diseases: in this case, it is the pain, that is wanting. Pain, you already know, is a symptom, that is absent in inflammations of the serous membranes, occurring in old persons, and in those whose nervous sensibility is impaired from a disorder of the brain. It is much more frequently absent in intense inflammations of the mucous membranes of the bowels, if stupor be present, for the natural sensibility of those membranes when inflamed is much below that of the serous tissue.

The constipation is the next symptom to be attended to. As a general rule, it denotes the absence of enteritis; indeed, gastro-enteritis rarely begins with the stomach, to pass thence down to the lower portion of the intestines; although, as you will see in severe cases of dysentery, the reverse is almost always true, and the disease extends upwards from the colon to the stomach. There was, likewise, in this case, no tension in the region of the stomach, a common sign of acute gastritis. Nor was there any pain on pressure, or tympanitis; so that the symptoms were, in a great measure, limited to the vomiting. The slightness of the pain may have been owing in part to the hæmorrhage, through which the inflammation found a natural outlet, which greatly relieved the severity of its symptoms, just as those of phthisis pulmonalis are relieved by a hemoptysis.

The coolness and pallor of the skin were directly connected with the inflammation of the stomach and the loss of blood. But the condition of the tongue was not characteristic of gastritis, being moist and very slightly coated. To the state of the tongue, during the prevalence of Broussaisism, there was attached a much greater importance, than it now receives, in diseases of the alimentary canal. While I was a resident physician at the Philadelphia Almshouse, the condition of the tongue was looked upon as a conclusive sign of the disease, and even of the degree and kind of inflammation. But, the experiments of Louis, Andral, confirmed by the researches of others, have since conclusively shown, that there is no necessary connection between the condition of the tongue and that of the alimentary canal. All that you can say is, that if the tongue is dry and red, your patient probably has gastritis; but the converse is not true; that is, if the tongue be clean and moist, it does not follow, that there is no gastritis. Besides, when it is red, dry, and chopped, there is generally something more than simple gastritis, as you noticed in a late case of dysentery, at the hospital, in which there was a very extensive inflammation of the mucous membrane of the alimentary canal. The black and coated tongue, covered with thick sordes, is nearly peculiar to those cases in which there is much depression of the nervous power, such as typhoid and typhus fever, &c. It arises, in part, from an altered state of the secretions, and partly from the patient lying with his mouth constantly open, so as to allow these secretions to dry upon the tongue and gums.

Now as to the diagnosis. With what diseases could the present be confounded? It could manifestly depend only on an affection of the stomach or liver. While the man was in a state of stupor, there was some doubt, whether the gastritis was acute or chronic, and whether it was secondary, depending on a cancer of the stomach, or primary, from intemperance. The treatment, however, was such as seemed necessary in acute gastritis occurring in a broken constitution, and would have been the same, whether it was acute or arising from cancer. It might, indeed, after the first few days of the patient's stay at the hospital, have been confounded with an affection of the liver, from the bilious vomiting, and discharge of blood, which might have taken place through the ducts of the gall-bladder. But, there was not fever enough to account for such an affection.

The next question is, was the affection of the brain primary or secondary? To solve it, we inquired into the habits of the patient; and a knowledge of them allowed us to infer, that the attack of epilepsy which occurred the day after his entrance, was produced either by the inflammation of the stomach, or by the direct vitiation or stimulation of the brain, from alcohol. The first was probably the true cause, and the affection of the brain was secondary to that of the stomach. You are aware how frequently the brain is affected, when the stomach is disordered, as in fevers, for example; now, in these cases, cerebral symptoms by no means necessarily depend on the condition of the stomach, but they form part of the train of symptoms, which constitute the disease, and often occur quite as soon as the gastric disorder. Here there was a different state of things, and the affection of the stomach was much more local, and not a mere symptom of a febrile disease. Another local cause of alteration of the cerebral functions is jaundice, as I have mentioned in a former lecture; the patient laboured under jaundice, as a consequence of the disorders of the stomach with which the liver soon sympathized, and the change in the qualities of the blood caused by jaundice, brings about a diminution of the intelligence, and especially of the memory.

Let us now examine the treatment of this case. Therapeutics are always difficult, when we come to reduce them to demonstration. Merely to prescribe is easy enough, and can be done by any old woman; but, to prescribe, so as to be certain that we will produce determinate results, is a very different affair. In fact, our remedies must often fail to produce the effects intended; and when such is the case, no *mauvaise honte* should prevent a frank confession of it. Remedies must, certainly, sometimes do harm, and it is a sacred duty in a teacher of medicine to point out every instance of such occurrences under his direction, with a manly disregard of the promptings of self-love and prejudice. I make these remarks, without particular reference to the present case; for we had nothing to complain of here, as each remedy administered produced the effect for which it was intended.

The treatment was very simple: for the brandy which the patient had been taking, we substituted at first other stimuli calculated to supply its place. We avoided using at first very powerful remedies, which should not be employed, unless clearly indicated, before the previous history of the patient is well ascertained. The remedies, at first directed, we found, however, not to be sufficient to meet the indications of the case. We then resorted to another most useful remedy, ice. The credit of introducing this article, as an ordinary therapeutic agent in the management of gastritis, belongs, I believe, to Broussais; at any rate, if before known, it was not, certainly, very generally employed with us, until fourteen years since, when the particular doctrines of Broussais became prevalent in this city. The remedies which were found of real utility have thus, you see, survived the exclusive doctrine which caused their introduction. Thus it is with many means of treatment, which are long known, and slightly used, but not common, until they are taken up by some individual; from the general employment of them dates their origin, not from the mere suggestion of them. The ice was taken by this man very freely; he was allowed to swallow as much of it as he found agreeable. Ordinarily, in gastritis, I use the ice in solution, giving iced water as cold as possible, in excessively small quantities, such as a tea, dessert, or table-spoonful, at short intervals. I enjoin upon the nurses never to leave the quantity to the patient's discretion, for even cold water, taken in large quantity, from mechanical distention, will increase to a high degree the irritation of the stomach, and cause its immediate rejection. Chewing ice is not, in general, so useful, as taking the iced water, for the ice is often retained in the mouth till melted, when it becomes warm by being entangled with the saliva and secretions of the mouth; whereas, the iced water passes down, at once, at a temperature of little more than 32°.

The next remedy applied was a blister. The propriety of blistering, in acute gastritis, is, I think, questionable, and I am not accustomed to resort to it, unless under peculiar circumstances. The use of blisters is strongly condemned by Broussais, in the acute form of the affection; but in the gastric fevers which were epidemic here from 1820 to 1828, the propriety of blistering was fairly tested, and the conclusion was very general, that it was injurious in the early stage of the fever, but, at the close of it, after vomiting is over, and the febrile movement not great, it answers a very good purpose. The blister was of good service in our case, although I cannot say precisely how much benefit was due to the ice, and how much to the blister, as they were employed together.

If this man had been a robust, stout, healthy subject, and had not presented a pale and prostrated aspect, the treatment would have been very different. We should have bled him largely, and afterwards leeched him, if he had suffered much pain on pressure in the region of the epigastrium; otherwise, we should have cupped him: if there is any considerable degree of pain, cupping is a very distressing application. Fomentations and poultices, the best of which is that of hops, are also to be applied to the pit of the stomach. The good effects of cupping, were illustrated in the epidemic of remittent and intermittent fevers, complicated with gastritis, which prevailed between 1820 and '28, to which I have alluded; but, latterly, the epidemics have changed their character, and it is not now often necessary to resort to the severe antiphlogistic regimen, that was indicated in fevers at the time of Broussais' observations. Broussais' observations were made in the north of Italy, the fevers of which region are accompanied by intense gastric inflammation, and his treatment of gum water, diet, and leechings, was very successfully applied to them. But when he afterwards attempted to manage the dothinenteritis of Paris, in the same style, his doctrine was no longer tenable.

Another remedy, used in the present instance, was brandy, a drachm of which was taken every half hour. This is often a very necessary remedy with drunkards, even if some gastritis be present; it may be given for a time, then suspended, and again returned to, if the patient be much prostrated. In this case, we found no other stimuli so good as brandy, and we accordingly returned to it, notwithstanding the aperient counter-indication of gastritis. The counter-indication is only apparent, for an organ long habituated to stimulation, recovers from inflammation with difficulty if all excitement be withdrawn for a considerable period.

Gastritis may be both primary and secondary. The primary type you have just had presented to you. Secondary gastritis of slight character often occurs in intemperate persons, as is the case with a man who entered the ward yesterday; he had drunk spirits till the powers of his stomach gave way. A slight gastritis, of this kind, is not to be treated by iced water, leeches, and the like, but with capsicum and carbonate of ammonia; in fact, pretty much as you manage delirium tremens: you change the nature of the stimulation, to which the patient is habituated, but you do not abandon it.

Gastritis is not a disease of great severity, recovery often taking place, even after considerable destruction of the mucous membrane. I had a preparation, which I made at Paris, of the stomach of a child, who once swallowed a quantity of a liquid, used at Paris in washing, a solution of indigo in sulphuric acid: the greater part of the stomach cicatrised, the child has perfectly recovered from the effects of the poison. You are not, therefore, to despair in any cases of severe gastritis, even in that produced by arsenic. When a poison of this kind is swallowed, as much danger is to be apprehended from its effects on the nervous system as from the local mischief in the stomach. I showed my class, last year, an excellent specimen of gastric inflammation, in the stomach of a man who committed suicide near Fairmount; he died from the effects of the arsenic, partly from the gastric symptoms, and partly from those of the brain. Softening of the mucous membrane, of which I have spoken as a sign of acute inflammation, was not present, as the arsenic had acted chemically upon it and hardened it.

The secondary gastritis which occurs in remittent fevers is very different from the primary affection. The confounding of the two was the great error of Broussais and the physiological school of medicine, every thing being treated by them as proceeding from the one source, irritation of the stomach. Broussais addressed his remedies to the gastritis solely, whereas the treatment of the gastric symptoms is now merely added to that required by the fever. The symptoms should, nevertheless, be carefully attended to; hence, if the gastritis be violent, the epigastrium is to be leeched, while you treat the symptoms of the brain by purging and cups to the head. This mixed practice is common in the West Indies, and is strongly recommended by Mr. Evans, who claims great success for it. During the day, he gives gum water, and applies leeches and poultices to the stomach, and at night he gives calomel, which, if given during the continuance of the more violent gastric symptoms, only increases the inflammation. The French, too, are now beginning to follow a mixed practice, particularly at Algiers, where, a late work, by an army physician, informs us, the malignant intermittents of the country were, at first, managed by low diet, gum water, and an exclusive antiphlogistic regimen. They were afterwards, however, managed with quinine, which was given even when there were symptoms of an acute character, indicative of gastric irritation, remaining throughout the interval between the paroxysms. The quinine was absolutely necessary to control the fever, and it was found that the local irritation was increased in a remarkable degree; the success was vastly greater. Such facts cannot be resisted, as they proceed from military surgeons, educated in the school of Broussais, and of course imbued with his doctrines. Broussais never uses purgatives, nor are the French, generally, as much in favour of them as the English. However, in the gastrites of fevers, we must admit the propriety of purging when the vomiting and pain are not excessive. If these symptoms are very violent, purging, particularly with the drastic remedies, will aggravate the disease, but when the gastritis is sub-acute there is no objection to mild cathartics. I have examined a good many patients, who have died from remittent fevers, and the cause of death I did not attribute to the inflammation of the stomach, but to the general symptoms of the disease. These fevers are cured by quinine—how, we cannot say, but must content ourselves with merely announcing the fact. Gastritis is a constant, necessary lesion in all these remittent and intermittent fevers, as far as we can judge from the symptoms during life, and the appearances after death. The gastritis, which is one of the anatomical lesions, is not the primary or essential disturbance. So also, in typhoid fever. No artificial gastritis ever produces the same affections of the brain and lungs that you meet with in these fevers of warm climates. The mucous membrane of the stomach may be entirely destroyed, and the patient recover without any such train of symptoms.

During the heat of summer we have many cases of gastritis from the effects of very cold water upon the stomach. These cases are confined to persons who have become excessively overheated and imprudently swallowed a large quantity of cold water, either iced or from the well. You have seen two such cases, both of which have recovered. We interrogated them carefully as to the circumstances under which their illness commenced. One had walked a considerable distance and drank largely of cold well-water; he immediately was seized with spasms at the stomach, followed by complete loss of consciousness. After he recovered his senses he was still giddy and feeble for a considerable time. The second was at work at the Exchange, carrying coals in one of the hottest days of the month. He was already languid and a little giddy in the morning, but at 11, A. M., drank about a pint of iced water; he immediately felt pain at the epigastrium with increased weakness and giddiness, and entered the hospital on the third day with a smart attack of gastritis.

Now you see the same cause occurring under the same circumstances, occasioned in the one patient decided gastritis, and in the other the affection of the stomach was much less than that of the brain. Both of these patients were in full health. The one in whom cerebral symptoms predominated had a slight valvular disease of the heart; the other was phthisical. A third case of very recent gastritis occurred at the beginning of the month, from drunkenness and exposure to the sun; the patient had not taken cold water. You may, therefore, understand from these cases that inflammation may arise from the direct effect of a stimulant, or from the reaction following a sedative.

Gastritis occurs as a secondary affection in almost all diseases in which there is much fever. Thus, the vomiting of the liquid like coffee-grounds, which takes place in acute phthisis, indicates the existence of gastritis, as has been observed by Dr. Louis. In typhoid, and, more rarely, in typhus fever, there is derangement of the stomach and of the brain, the sympathies of which are more various than even those of the stomach. In chronic phthisis there are often gastric symptoms. If there be severe pain and sensation of heat at the liver and epigastrium, the treatment is to be addressed to the stomach; you must stop the administration of porter, or other stimulating articles, and confine the patient to milk and farinaceous diet, but you are not to treat the case as idiopathic gastritis, or resort to more energetic means than strict abstinence.

For a complication of gastritis with pneumonia, no particular treatment is demanded, except that the propriety of giving tartar emetic for the latter affection then becomes questionable. Tartar emetic differs from other poisons, in attacking the follicles of the mucous coat of the alimentary canal. The inflammation of the follicles thus produced in pneumonia might be mistaken for that following dothinenteritic, but is to be distinguished from it by the subjacent cellular coat of the follicles not being attacked, also by the nearness of the affection to the stomach, and by the absence of those alterations of the spleen and mesenteritic glands, so constant in typhoid fever. In the administration, therefore, of high doses of tartar emetic in pneumonia, say half a grain every two hours, you must remember that inflammation of the follicles may ensue; and you must be particularly careful if you have any considerable degree of stupor, as it may mark this variety, gastro-enteritis.

The connection between gastritis and enteritis I shall enter upon, at a future lecture.

---

DYSENTERY.

*Tuesday, July 10th.*—I have been for a long time designing to address you on the subject of dysentery, but have preferred waiting, until a case illustrative of it should present itself. We have now a case in the wards, and as, owing to the season of the year, and the heat of the weather, diseases of the alimentary canal will no doubt be soon unusually rife, I cannot do better than enter now upon this topic. In a preceding lecture, I occupied your attention with the subject of acute gastritis, and the natural passage from this subject would have been to that of chronic gastritis; but inasmuch as it is very obscure in character, and there are diseases now prevalent, of a more active nature, I prefer postponing it to the latter end of my course.

We shall proceed to the subject of inflammation of the alimentary canal. The first illustration I shall bring before your notice, is that of a man who died a few weeks ago in the lunatic department of the almshouse. He had a dysentery, which had remained unchecked for six or seven days, and presented at the time I saw him, the symptoms of excessive pain, involuntary stools, discharges very fœtid and containing blood, with a thin, dark liquid, parched and dry tongue, great thirst, anorexia, and excessive feebleness. His history could not be learned, from the condition of his intellect. The treatment, in his case, was limited to opium and slight mercurials. The fatal character of the affection I predicted from the first, and I have instanced it, particularly, to call your attention to the fact that lunacy predisposes to death, in dysentery, with astonishing force. As the symptoms are necessarily, to a degree, latent, they are less amenable to art.

The next case I shall mention, is a fatal one that occurred in May last; not, however, in my own ward. The symptoms, at the time of the man's admission, were involuntary, fœtid, and bloody stools, dry tongue, and frequent, feeble pulse. He died a few hours afterwards, having taken opium, with a small quantity of ipecacuanha and calomel. After death, we found in the large intestine, large rounded ulcerations, with disease of the follicles, the intervening mucous membrane swollen, and of a deep purple tint, as occurs, between the ulcerations, in all severe dysenteries, when the whole mucous membrane is not involved. But, in a more advanced stage of the disease, the ulcerations are vastly more extensive; they begin in the follicles, but afterwards run together, so that, in very bad cases, the whole circumference of the colon becomes one vast ulcer, while, in the cœcum and rectum, the ulceration is confined to the follicles. It is most rapid and proceeds with the greatest violence, in the sigmoid flexure and transverse colon.

The cause of the ulceration lies always in disease of the follicles. If a patient die at an early period of the affection, you can detect in each follicle a distinct, rounded ulcer, with a deep, central point, of a dark colour. Usually, the follicles are redder than the rest of the mucous membrane, and seem to be in a more acute state of inflammation. These are the appearances, common in dysenteries, of a moderate type. When it is of a more severe grade, the inflammation is of a different character. The intervening mucous membrane between the follicles, instead of being swollen and red, is livid, blackish, and presents a sphacelated appearance. This occurs, to a considerable extent, when dysentery is epidemic, as was the case last year, in my ward, at the hospital, when the mucous membrane of nearly all the bowels, which we examined, was in a sphacelated condition. This is the worst form in which the disease can show itself, and if you ascertain that it exists, during life, as you can usually do, by the gangrenous fœtor of the dejections, you may expect a fatal termination.

Another variety of anatomical lesion occurs in dysentery, but rarely except in that of the insane, and in that which sometimes complicates measles. There is deep inflammation of the mucous membrane of the colon, which is of an intensely red or purple colour, with patches of false membrane, spread over its surface, like a coating of mortar; if the inflammation reach the small intestine, they follow the shape of the valvulæ connirentes, which appear quite incrusted with it. These granules show themselves, wherever they have not been destroyed by the ulceration. The prognosis is here very severe; but, of course, the condition of the intestines can be merely conjectured, from the symptoms. If patches of false membrane are thrown out, with blood, from the bowels, the inference is plain enough: this type of the affection is, however, less dangerous than the sphacelated.

These are the anatomical characters of all the varieties of dysentery. In severe epidemics, you must bear in mind, however, that you will meet with a number of cases, where the morbid phenomena will differ in intensity, from what the symptoms had induced you to expect. Again—although the colon is to be considered as essentially the seat of dysentery, there will be other symptoms, independent of this local cause, owing to the extension of the disease, and its impression upon the nervous system. The symptoms, connected with the extension of the disease along the course of the alimentary canal, are those which involve the stomach. They are thirst, strong desire for drinks, and intense vomiting: towards the latter stages of the disease, if these symptoms are combined with a dry, red tongue, they are to be considered grave, and the patient will resist all medication, because the stomach will retain neither medicine nor food. The nervous symptoms, in some cases, are not, it would seem, connected, with the state of the colon, being often intense, while the lesion of the latter is very slight. In epidemics, you will often have a severe development of nervous symptoms, and, after death, you will find nothing in the alimentary canal, to account for them. The disease cannot, in these cases, be merely of the intestines, but must be, therefore, in part at least, nervous, depending on some cause acting on the whole system.

The present patient has been ill, since the fourth of July. He is a native of Germany, has been five years in this country, and is of temperate habits. Since he was taken ill, he has been constantly getting worse, passing blood, since the fifth, in increased quantities on the sixth, and afterwards. He has had constantly severe pain, much increased for the last two days. He has had great thirst, but abstained from drinking, thinking it would do him harm. Appetite almost lost; no vomiting. He took some draught at an apothecary's, the nature of which he does not know.

His present state is the following:—Air of exhaustion, face reclining to one side, lips a little dark, face slightly flushed, dull look. Tongue coated with a yellowish fur, reddish at the edges, and rather dry. Thirst, anorexia. Stools incessant, six or seven in the last few hours, a little very thin mucus in the last stool, blood in the preceding stools. Abdomen rather full, very tender every where, especially over the transverse colon. Pulse eighty-four, soft and full; voice a little fluted in tone. This fluted tone of the voice is by no means rare in affections of the alimentary canal, and is an almost constant symptom in Asiatic cholera.

The man had no cough nor eruption on the skin. The discharges were tested, and the feces and perspiration were found very alkaline, and the saliva, slightly so. The urine alone remained slightly acid, so that the acid character of the perspiration was totally lost, and that of the urine was very much diminished.

The symptoms here, then, are evidently those of dysentery. It is a disorder which may attack the patient either suddenly or some time after the occurrence of a diarrhœa, usually the latter, some interval elapsing between the appearance of the simple diarrhœa and the dysentery, just as bronchitis passes into pneumonia. As soon as the disorder attains the point of dysentery, the stools diminish in quantity, are voided with pain, contain some tenacious mucus and lose their fœtor. The pulse becomes small and quick. The other symptoms are not quite so regular in their occurrence. There is generally pain in the abdomen, most intense in the region of the transverse colon, though not confined to this spot. There is great thirst, the appetite is lost in severe, though not entirely in mild cases. There are well marked cerebral symptoms in many cases, although they are by no means universal. There is never any alteration of the intelligence during the height of the affection, as in fever; when it occurs, it is always at its end, and is brought about by nervous prostration; whereas, in typhoid, bilious, and remittent fevers, it is an early and principal symptom. There may be in dysentery, extreme sighing, subsultus, and insomnia, but very rarely loss of memory. In this case, there were merely prostration, disgust for food, and the fluted tone of the voice, in addition to the abdominal symptoms. This fluted tone, occasional in dysentery, and very common in Asiatic cholera, is a hard and shrill sound, and is not to be easily confounded with the hoarseness of laryngitis.

The diagnosis, in dysentery, offers no difficulty. It is so local an affection, and so near the exterior of the body, that, at a glance, it is recognised. When the disease is acute and epidemic, there can be no difficulty about distinguishing it; this can only occur when it is chronic or sporadic. You must be careful not to confound it with febrile or cerebral affections. The only distinction necessary in regard to the disease itself, is to divide it into its primary and secondary forms. The secondary form of dysentery is not common in winter, but occurs, during the summer, when it is epidemic; for, it impresses its character upon all diseases. Thus, in typhoid fever, there is, naturally, a very slight diarrhœa; but, when dysentery is epidemic, this becomes severe, with bloody, painful stools. Two years ago, when there were a number of cases of typhoid fever, during an epidemic of dysentery, I lost two patients, who were convalescent from the fever, but who, having been left in a weak state, were carried off by an attack of dysentery. In typhus fever, it is more difficult to estimate how much of the dysentery is owing to the typhus, and how much is independent of this cause. Last summer, when the epidemic of typhus was just ending, scarcely a case terminated without severe dysentery.

Patients, who are liable to frequent attacks of diarrhœa, as tubercular subjects, for example, may have attacks of dysentery, which may be very readily distinguished from their ordinary diarrhœa. Last year, I had several tuberculous patients, liable to slight diarrhœas, who were seized with dysenteries of intense severity, which, from their violence, could not be confounded with the tubercular affection.

Chronic dysentery is more difficult of diagnosis, as the term embraces a number of distinct dysenteric affections. In some places, it is applied to acute dysenteries, become chronic; in other cases, there is no preceding acute affection, but the chronic disorder begins abruptly. In dysenteries, strictly chronic, to say where there is decided ulceration of the mucous coat of the bowels, and where there is mere alteration of its secretions, is sometimes a very puzzling matter. I had a case, some time since, in my ward, of a man, who laboured under a chronic dysentery, for eight months, before he entered the hospital. We tried a variety of treatment for his relief,—but he sank, presenting, some time before his death, tubercular symptoms. On examination after death, we found a few tubercles in the lungs, and the mucous membrane of the intestines softened, of a light grayish tint, rather thicker than naturally, but without false membrane or cicatrices. Had this dysentery been preceded by an acute affection, we should have ulcerations or cicatrices. This I call chronic dysentery, distinguishing it from that succeeding the severe, acute affection, with ulcerations. If a patient is moderately intelligent, it is always easy to pass back to the first invasion of the disease, and to make the distinction I have mentioned.

I have detailed to you the anatomical characters of the severe type of dysentery. But, if the patient recover, and, during his convalescence, is carried off by an accidental and independent affection, you can trace the anatomical marks that distinguish the progress of the dysentery towards a cure. The edges of the ulcerations are sunk down, less red, of a more or less slate colour, in place of the purple and dark livid hue. The bottom of the ulcer is no longer the muscular or cellular coat of the intestine, but is lined with a new membrane. Cicatrices may be known by their bluish tint, smoothness, and their being below the natural level of the membrane. In a remarkable case, I found cicatrices throughout the whole colon, a case, which had passed from the acute to the chronic form, of thirteen months' standing. From this, you may infer, that, no matter how severe sloughing or hæmorrhage may be, the patient may get well, if his constitution be good, and the mucous membrane of his bowels may be restored to a degree of cicatrization, quite consistent with health. The anatomical lesions at the close of dysentery, are the development of tubercular depositions, and the passage of the acute into the chronic form. The ulceration rarely runs into perforation. For a proper understanding of the affection under notice, you see how important is a knowledge of pathological anatomy, and how impossible it is to estimate the chances of a cure, without an acquaintance with the results of this branch of medical investigation.

Another set of symptoms, connected with dysentery, is the chemical alteration, that takes place in the secretions of the body. An impetus has lately been given to this branch of investigation, that promises to lead to important results. The experiments of Donné on this subject are interesting. Last spring, I requested one of my pupils, Mr. Griscom, to take up the subject of the secretions in dysentery, and he produced an experimental inaugural thesis, which contained some valuable contributions to medical science, which I shall take a future occasion to communicate to you. In the case to-day, we tested the secretions, and found the feces, saliva, and perspiration very alkaline, and the urine only feebly-acid, showing a strong preponderance of alkaline matter. I followed out an indication, which seemed here to offer itself, and gave sulphuric acid, in doses of ten drops, three times a day. A poultice was placed over the abdomen, with a drachm of laudanum in it. Should this treatment fail, I shall modify the treatment, and place the man on other remedies, which prove generally successful, as the various preparations of opium; ipecacuanha, alone, or combined with mercurials; mercurials, as purgatives, followed by castor oil, or the oil alone; or depletion to the anus or abdomen.

In my next lecture, I shall enter, at length, into the various modes of treatment, employed in dysentery.

